# Determination of Equilibrium and Kinetic Parameters of the Adsorption of Cr(III) and Cr(VI) from Aqueous Solutions to *Agave Lechuguilla* Biomass

**DOI:** 10.1155/BCA.2005.55

**Published:** 2005

**Authors:** Jaime Romero-González, Jorge L. Gardea-Torresdey, José R. Peralta-Videa, Elena Rodríguez

**Affiliations:** 1 Environmental Science and Engineering Ph.D. Program University of Texas at El Paso El Paso TX 79968 USA; 2 Department of Chemistry University of Texas at El Paso El Paso TX 79968 USA

**Keywords:** Adsorption, Cr(III), Cr(VI), kinetic, equilibrium, lechuguilla

## Abstract

This investigation reveals the capability of *Agave lechuguilla* for trivalent and hexavalent chromium removal from aqueous solutions. Experimentation included pH profile, time dependence, adsorption capacity (K_F_ and Q_L_), adsorption intensity (*n* and R_L_) and saturation capacity (*q*_s_) studies. Batch experiments were conducted at 22^∘^C to characterize and model the adsorption equilibrium as well as biomass adsorption rates. pH 4 was the optimum for Cr(III) binding, while Cr(VI) optimum binding was at pH 2. Time profile experiments indicated that the adsorption of Cr(VI) by lechuguilla biomass was time-dependent and that of Cr(III) was not. Kinetic models demonstrated that a pseudo-second order reaction model best described the kinetic data for Cr(VI). The adsorption isotherms showed that the binding pattern for Cr(VI) followed the Freundlich isotherm model, while that for Cr(III) followed the Langmuir isotherm.


### Determination of Equilibrium and Kinetic Parameters of the Adsorption of Cr(III) and Cr(VI) from Aqueous Solutions to *Agave Lechuguilla* Biomass.

Jaime Romero-González[1], Jorge L. Gardea-Torresdey [1,2*], José R. Peralta-Videa[2], Elena Rodríguez[1]

[1]*Environmental Science and Engineering Ph.D. Program and* [2]*Department of Chemistry, University of Texas at El Paso, El Paso, TX 79968, USA*

### ABSTRACT

This investigation reveals the capability of *Agave lechuguilla* for trivalent and hexavalent chromium removal from aqueous solutions. Experimentation included pH profile, time dependence, adsorption capacity ($K_F$ and $Q_L$), adsorption intensity ($n$ and $R_L$) and saturation capacity ($q_s$) studies. Batch experiments were conducted at 22°C to characterize and model the adsorption equilibrium as well as biomass adsorption rates. pH 4 was the optimum for Cr(III) binding, while Cr(VI) optimum binding was at pH 2. Time profile experiments indicated that the adsorption of Cr(VI) by lechuguilla biomass was time-dependent and that of Cr(III) was not. Kinetic models demonstrated that a pseudo-second order reaction model best described the kinetic data for Cr(VI). The adsorption isotherms showed that the binding pattern for Cr(VI) followed the Freundlich isotherm model, while that for Cr(III) followed the Langmuir isotherm.

**Keywords:** Adsorption, Cr(III), Cr(VI), kinetic, equilibrium, lechuguilla

### GRAPHICAL ABSTRACT

The biosorption of Cr(VI) and Cr(III) from dilute metal ion solutions by *Agave lechuguilla* biomass demonstrated that this biomass has the potential of being used to clean up Cr-contaminated water. In addition, these studies will contribute to the understanding of the mechanism and rate of adsorption of these metal ions.

* Corresponding author: Tel: +1-915-747-5359; Fax: +1-915-747-5748; e-mail: jgardea@utep.edu

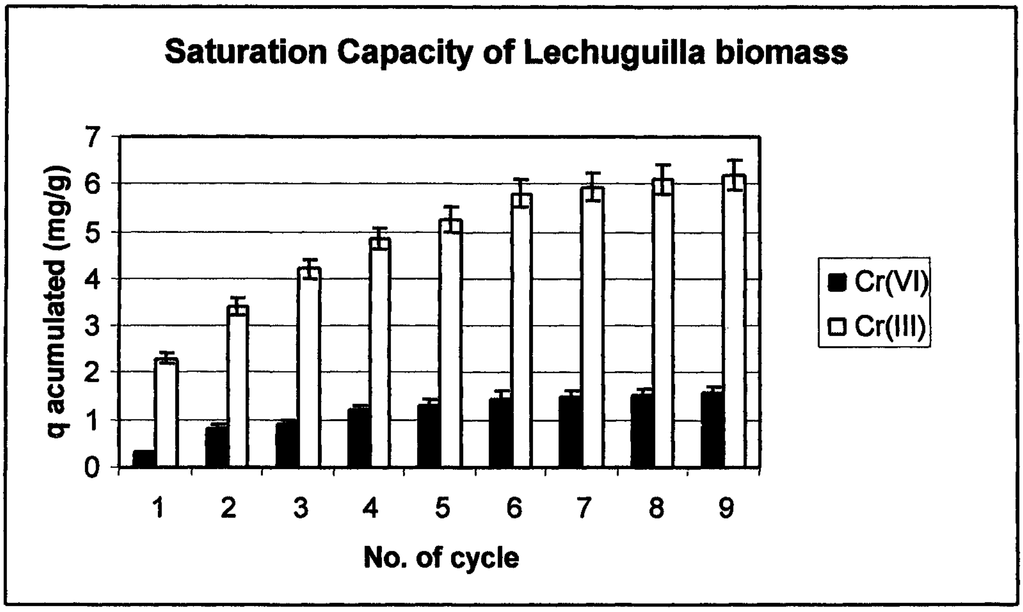

### 1. INTRODUCTION

Chromium is very common in the environment and can be found in concentrations ranging from less than 0.1 $\mu g/m^3$ in air to 4 g/kg in soil /1-4/. Naturally occurring chromium is in the trivalent state in rocks, soil, plants, and volcanic emissions. Hexavalent chromium is mostly derived from industrial activities such as steel making, electroplating, and tanning industries /3-6/. Figure 1 shows the environmental cycling, the sources, and oxidation states of chromium.

The physiological effects of chromium on biological systems depend on its oxidation state. At low concentrations Cr(III) is considered an essential element in mammals for the maintenance and control of glucose, lipid, and protein metabolism. However, at the same low concentrations Cr(VI) is toxic. Its effects on living systems due to its tendency to oxidize other chemical species have proven to damage the lungs, liver, nervous system, and kidneys in mammals /3/. The U.S. Environmental Protection Agency (USEPA) has set the maximum contaminant level for Cr(III) and Cr(VI) at 0.1 mg/l. However, other national and international drinking-water associations such as the California Department of Health Services (CDHS) and the World Health Organization (WHO) reject drinking-water containing chromium above 0.05 mg/l /7,8/.

Chromium is found as chromium(III) and chromiumCr(VI) in aqueous phase. Trivalent chromium exists as hexa-aquachromium, $Cr(H_2O)_6^{3+}$, as well as aquahydroxo products such as $Cr(OH)^{2+}$, $Cr(OH)_2^{+}$, $Cr_3(OH)_4^{5+}$, and $Cr(OH)_3$ /9/. Hexavalent chromium may exist in the aqueous phase in different oxyanionic forms such as chromate ($CrO_4^{2-}$), dichromate ($Cr_2O_7^{2-}$), or hydrogen chromate ($HCrO_4^-$) /10/.

The following equation shows the relation between hexavalent and trivalent states of chromium /11/:

$$Cr_2O_7^{2-} + 14H^+ + 6e^- \rightarrow 2\,Cr^{3+} + 7H_2O \qquad E_o = 1.33v.$$

Due to the health hazards of chromium, numerous studies concerning its removal from aqueous solutions have been performed using different biomasses /12-18/. However, few studies have compared the

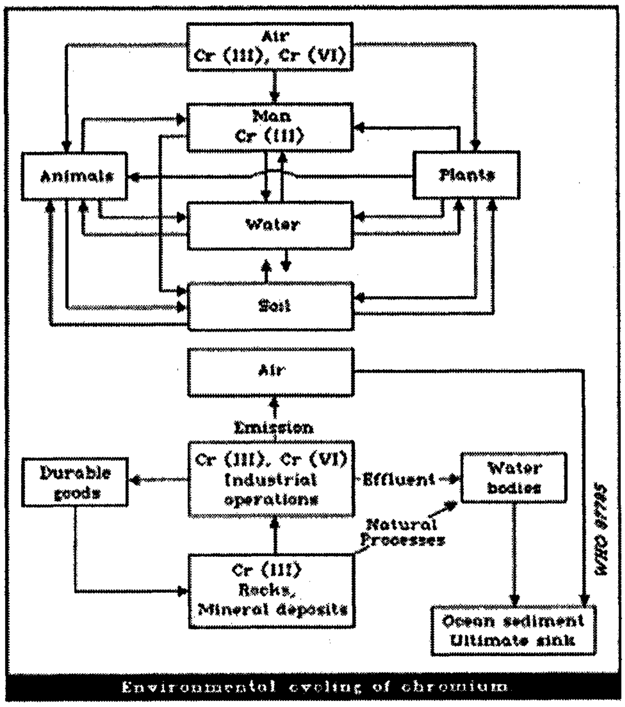

**Fig. 1:** Environmental cycling of chromium.

equilibrium and kinetics parameters of the adsorption of both oxidation states with the same biomass /19-20/.

*Agave lechuguilla*, commonly known as lechuguilla, is one of the most common plants of the Chihuahuan Desert as well as other desert areas in Mexico and the U.S. (Figure 2). Lechuguilla is a small plant consisting of a few-leaved rosette about 30-40 cm long. It grows on rocky limestone slopes. The number of individual rosettes (average of 21,000 per hectare) probably exceeds all other native agaves. The plants accumulate nutrients during the growth period and die after flowering, leaving an abundance of cellulosic biomass /21/. These characteristics make lechuguilla an attractive biomaterial for the removal of heavy metals from water and wastewater.

The objective of this study was to investigate the equilibrium and kinetic parameters of the adsorption of trivalent and hexavalent chromium from aqueous solutions by lechuguilla biomass. The parameters studied included pH profile, time dependence, adsorption capacity, adsorption intensity, and saturation capacity. Batch experiments were conducted to characterize and model the adsorption equilibrium as well as the adsorption rates. The results of these studies will contribute to the understanding of the mechanisms and adsorption rate of chromium ions.

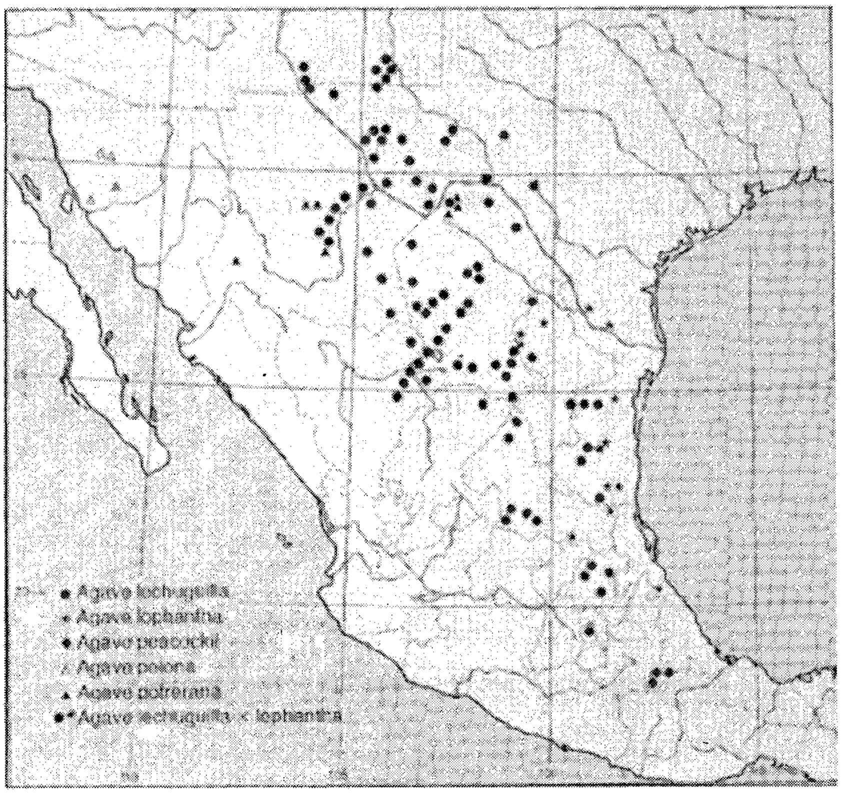

**Fig. 2:** Comparative distribution of *Agave lechuguilla*.

### 2. EXPERIMENTAL

### 2.1 Lechuguilla collection

Several dead lechuguilla plants were collected in the vicinity of El Paso, Texas. The plants were washed thoroughly using tap water in order to remove any soil or debris. Only the leaves of the plants were utilized in this study since they represent more than 90% of lechuguilla plants. The washed samples were oven dried at 80° C for 3 days and ground to pass through a 0.150 mm sieve using a Wiley mill.

### 2.2 Metal analyses

A flame atomic absorption spectrometer (FAAS) (Perkin-Elmer model 3110) was used to determine the total chromium content in plant samples. The analytical wavelength used was 359.4 nm with a slit width of 0.7 nm. The chromium hollow-cathode lamp current was 30 mA. An impact bead was used to improve instrument sensitivity. Standards were prepared by dilution of a 1000 mg/l stock solution and calibration curves were obtained using 6 points including the blank. Correlation coefficients ($r^2 = 0.98$ or better) of the linear calibration curves were obtained.

The samples were run in triplicate and the mean value and relative standard deviation were recorded. In order to work within the linear calibration range, some samples were diluted using 5% $HNO_3$. The final metal concentration was subtracted from the initial metal concentration and the difference was assumed to be the amounts of chromium (either Cr(III) or Cr(VI)) adsorbed by the lechuguilla leaf biomass.

### 2.3 pH profile studies for metal binding

This experiment was carried out using the pH profile method previously reported by Gardea-Torresdey *et al.* /22/. A 250-mg sample of lechuguilla leaf biomass was washed four times with 0.01 M HCl using a centrifuge to remove any debris or metals ions from the biomass. The sample was then washed three times with DI water in order to remove soluble material or biomolecules that might interact with any sorbed metal ions. The washed biomass was resuspended in 50 ml of DI water to obtain a concentration of 5 mg of lechuguilla per ml of water. The suspension was adjusted to pH 2 using diluted solutions of HCl and NaOH. Six aliquots of 4 milliliters each of the biomass suspension were placed into six clean test tubes. The tubes were agitated with a rocker for 60 min and then centrifuged at approximately 3000 rpm for 5 min (Fisher Scientific Marathon K 8). The biomass pellets were separated from the supernatants and saved for the next experiments. Separate 0.1 mM solutions of Cr(III) or Cr(VI) were prepared from the corresponding salts, $Cr(NO_3)_3$ and $K_2Cr_2O_7$, and adjusted to pH 2. Three 4-ml aliquots of Cr(III) or Cr(VI) solution were transferred to the test tubes containing the lechuguilla biomass pellets. Three more 4 ml aliquots were transferred to clean test tubes and set as control. The tubes were then rocked for 1 h and centrifuged. Similar procedure was followed for each of the following pH values: 3, 4, 5 and 6. The final pH of the supernatants was recorded and the chromium content was determined using flame atomic absorption spectroscopy. Each experiment was performed in triplicate for quality control and statistical purposes.

### 2.4 Time dependence for Cr(III) and Cr(VI) binding

The time dependence experiments were performed in a similar fashion to that previously reported by Gardea-Torresdey *et al.* /22/. A 250 mg sample of biomass was washed in DI water in order to remove any metal ions or soluble materials that might interfere with Cr adsorption. The biomass was then re-suspended in 50 ml of DI water to obtain a final biomass concentration of 5 mg/ml. The biomass suspension was then adjusted to the appropriate optimal pH, determined from the pH profile studies: pH 2 for Cr(VI) and pH 4 for Cr(III). Aliquots of 4 ml of 0.3 mM metal solution (Cr(VI) at pH 2 or Cr (III) at pH 4) were added to the 42 tubes (21 per Cr ion) containing biomass pellets and allowed to react for: 5, 10, 15, 30, 60, 90 and 120 min. At each time interval the test tubes were centrifuged and the supernatants were discarded. Three additional tubes containing Cr(III) or Cr(VI) solution were maintained as control for each time period. The tubes (containing approximately 20 mg of biomass and 4 ml of 0.3 mM metal solution and the respective controls) were then rocked for their respective time interval and then centrifuged at 3,000 rpm for 5 min. The same procedure was followed for both metals being studied at their optimal binding pH. The supernatants from all of the pellets were transferred to clean test tubes and analyzed for chromium content using FAAS.

### 2.5 Saturation adsorption capacity for lechuguilla biomass

For the saturation adsorption capacity studies, three 4-ml of biomass solution (5 mg/ml) were taken and transferred to clean test tubes. The biomass was reacted with four milliliters of 0.3 mM Cr(III) or Cr(VI) solution at the predetermined optimal binding pH. The biomass was reacted with the metal solution for 15 min using a rocker. After that, the samples were centrifuged at 3000 rpm and the supernatants were saved for metal analysis. This procedure was repeated nine times or until the biomass was saturated with Cr ions. After the nine reaction cycles, four ml of 0.1M HCl were added to the biomass and allowed to react for 15 min in order to desorb the bound metal ions. Subsequently, the samples were centrifuged and the supernatants were retained for further metal analysis. This procedure was repeated until no Cr was detected in the supernatant.

The saturation adsorption capacity is reported in mg/g and the amounts of metal removed from the biomass (desorption) are given in percentages. Three replicates per treatment were run for each experiment.

### 2.6 Adsorption capacity and affinity

The adsorption capacity and affinity of lechuguilla for Cr removal was determined with different models of isotherms using Cr(VI) and Cr(III) solutions at 10, 15, 20, 25, 30, 35, and 40 mg/l. These experiments were performed following the procedure described for the time dependence studies. In summary, aliquots of 4 ml of each Cr solution were added to biomass pellets and allowed to react for 12 h in order to obtain equilibrium conditions.

### 3. RESULTS AND DISCUSSION

### 3.1 pH profile

The percentage binding of Cr(III) and Cr(VI) to lechuguilla biomass are shown in Figure 3. As one can see in this figure, the binding of both Cr(III) and Cr(VI) ions is pH dependent. However, the amount of Cr(III) bound to lechuguilla biomass increased as pH increased. At pH 4, lechuguilla showed a maximum binding of 94% of the Cr(III) ions present in the solution. Other researchers found that at pH 4 different adsorbents showed maximal Cr(III) removal in a range from 16 % to 99 % /23,24,25/. On the other hand, the binding of Cr(VI) to lechuguilla biomass decreased as pH increased, showing the maximum (34%) at pH 2. The removal percentages reported ranged from 19.8 to 69.3 %, at pH 2 /25/. This trend in pH dependence suggests that the binding of the metals to the biomass is through an ion exchange mechanism. Cr(VI) exists in solution predominantly as an anion ($HCrO_4^-$) at low pH values and as $CrO_4^{2-}$ at high pH values. Cr(III) tends to form cationic hydroxides in solutions such as $Cr(OH)^{2+}$, $Cr(OH)_2^+$, and $Cr_2(OH)_4^{2+}$.

The adsorption of cations and anions to surfaces can be described by the following reactions according to a surface-binding model:

$$B\text{-}OH \leftrightarrow B\text{-}O^- + H^+ \qquad (I)$$

$$B\text{-}OH + H^+ \leftrightarrow B\text{-}OH_2^+ \qquad (II)$$

$$B\text{-}O\text{-} + C^+ \leftrightarrow B\text{-}OC \qquad (III)$$

$$B\text{-}OH_2^+ + A^- \leftrightarrow B\text{-}OH_2A \qquad (IV)$$

where B represents the biomass, B-OH represents a typical surface functional group such as carboxylic ligands found in the cell walls of the biomass, $C^+$ and $A^-$ represent a cation and an anion, respectively. These complex ion reactions are highly pH dependent because of the extent of surface deprotonation, (reaction I), and protonation, (reaction II) which is controlled by solution pH. The solution pH at which the surface of an adsorbent particle carries no charge is called the pH of point of zero charge ($pH_{pzc}$). If the solution pH is greater than $pH_{pzc}$, the surface is negatively charged, which would allow more cation adsorption. When the pH is less than $pH_{pzc}$, the surface is positively charged, which allows more anion adsorption. To calculate the value of the $pH_{pzc}$ for lechuguilla biomass, surface charges were measured by potentiometric tritrations of solutions containing 5 mg/ml of biomass in 0.01, 0.1 and 1 M NaCl, using 0.1 M HCl as titrant. The values of $pH_{pzc}$ for lechuguilla biomass were estimated from the arithmetic average of $pK_1$ and $pK_2$ values /27/. The $pH_{pzc}$ for lechuguilla occurred between 2.42 and 2.71, with an average of 2.58 (Table 1). It has been reported that the carboxyl functional groups of algal cell walls have a $pH_{pzc}$ value of 2.6 /26/. At pH values above

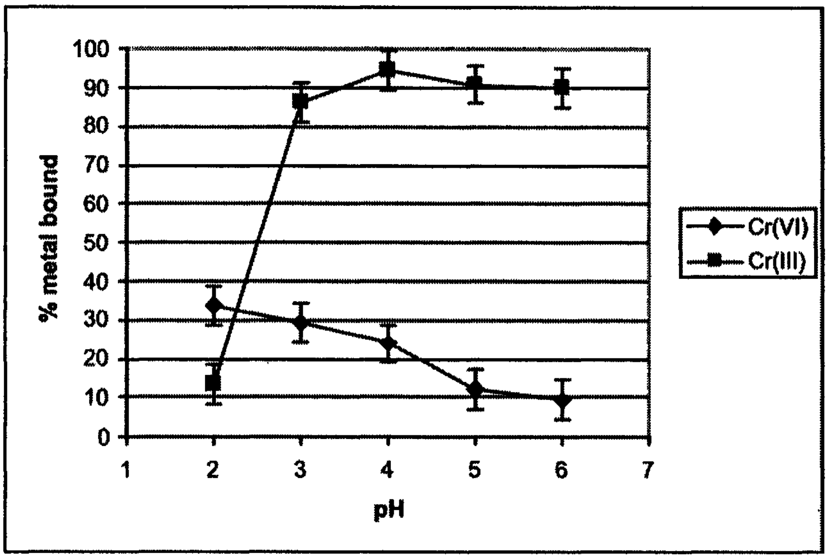

**Fig. 3:** pH profile for Cr(VI) and Cr(III) by lechuguilla biomass. Experiments were performed with chromium solutions 0.1mM concentrated.

**Table 1.**
Intrinsic acidity constant of lechuguilla biomass.

| C (M NaCl) | $pK_1$ | $pK_2$ | $pH_{pzc}$ |
|---|---|---|---|
| 1 | 3.7 | 1.72 | 2.71 |
| 0.1 | 2.9 | 1.95 | 2.42 |
| 0.01 | 3.2 | 2.03 | 2.61 |
| | | $(pH_{pzc})_{average}$ | **2.58** |

2.58, the biomass carries an overall negative charge; consequently, the adsorption of Cr(VI) anions is expected to decrease as pH increases. The pH dependence of Cr(III) and Cr(VI) binding observed in this study is consistent with the proposed surface-binding model.

### 3.2 Adsorption kinetic parameters

The amounts of Cr(III) and Cr(VI) bound to lechuguilla biomass resulting from the time dependence studies are shown in Figure 4. As one can see in this figure, the process of Cr(VI) adsorption by lechuguilla is time-dependent while Cr(III) is not. The trend in Cr(III) adsorption suggests that the binding of this ion may be through interactions with functional groups located on the surface of lechuguilla biomass. However, the mechanism of Cr(VI) binding is different. This difference could be due to the reduction of Cr(VI) to aquahydroxo Cr(III) complexes. These complexes have a strong tendency to be adsorbed by naturally occurring solids, which contribute to decrease mobility of Cr(III) in water. Cr(VI) species, on the other hand, are only weakly adsorbed, being the most mobile form of chromium in the environment /3/.

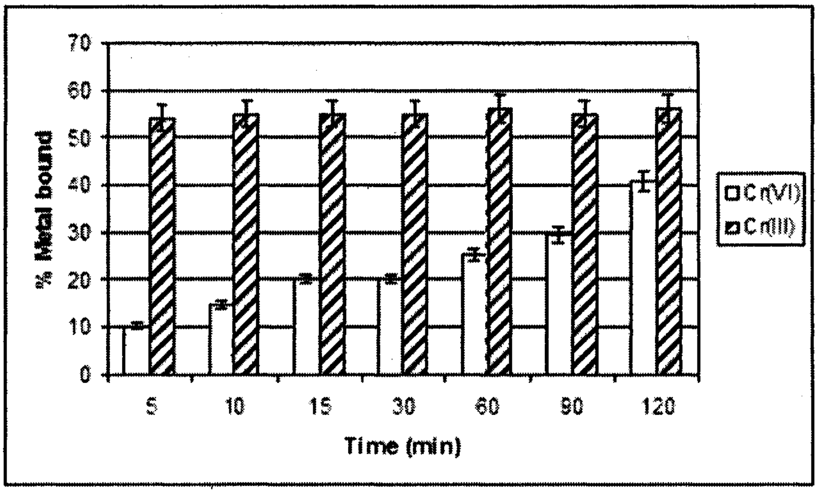

**Fig. 4:** Time profile for Cr(III) and Cr(VI) binding by lechuguilla biomass. Experiments were performed with chromium solutions 0.3mM concentrated. Error bars indicate 95% normalized confidence interval.

### *3.2.1 Kinetics models*

To investigate the mechanism of Cr(VI) biosorption, the experimental data of the time dependence studies were utilized in the first-order and pseudo second-order kinetic models.

The first-order rate expression of Lagergren /28/ based on solid adsorption capacity is generally expressed as follows /29/:

$$dq/dt = K'_{ad}\,(q_e - q) \tag{1}$$

where $q_e$ is the amounts of solute adsorbed at equilibrium per weight of adsorbent (mg/g), $q$ the amount of solute adsorbed at any time (mg/g), and $K'_{ad}$ is the rate constant of first-order biosorption ($min^{-1}$). If equation (1) is integrated for the boundary conditions $t$=0 to t >0 and $q$=0 to $q$>0, the following linear time dependence function is obtained:

$$\text{Log}\,(q_e - q) = \log(q_e) - (K'_{ad}/2.303)\,t \tag{2}$$

The experimental data obtained from the time dependence study was used in equation (2). The results of the appropriate calculations are shown in Figure 5. The data shown in this figure were used to estimate the constants shown in Table 2.

The pseudo-second order model /30/ is also based on the sorption capacity of the solid phase. The pseudo second-order chemisorption kinetic rate equation is expressed as the following:

$$dq/dt = K''_{ad}\,(q_e - q)^2 \tag{3}$$

where $K''_{ad}$ is the rate constant of second-order biosorption (g $mg^{-1}$ $min^{-1}$), $q$ and $q_e$ represent the variables explained before. Integrating equation (3) for the boundary conditions $t$=0 to t>0 and $q$=0 to q>0, the

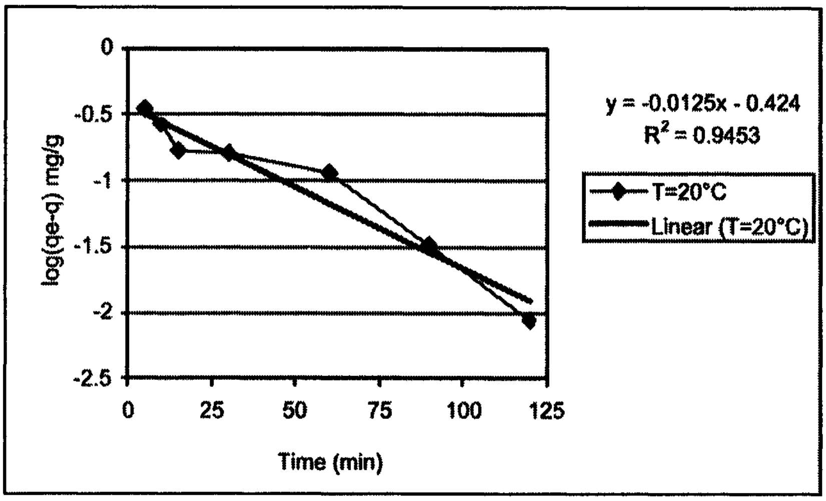

**Fig. 5:** Linear plot of first order reaction for the adsorption of Cr(VI) to lechuguilla biomass.

following linear time dependence function is obtained:

$$t/q = 1/2\ K''_{ad} q_e^2 + (1/q_e)\ t \quad (4)$$

The experimental data obtained from the time dependence study was used in equation (4). The results of the appropriate calculations are shown in Figure 6. The data shown in this figure were used to estimate the constants shown in Table 2.

**Table 2**

Comparative analysis of linear pseudo first order and pseudo second order reaction rate equations, their constants and correlation coefficients ($R^2$ values), for the adsoprtion of Cr(VI) to lechuguilla biomass.

| Model type | Constant of pseudo reaction | $q_e$ (mg/g) | $R^2$ |
|---|---|---|---|
| Pseudo first order | $K'_{ad} = 0.028\ min^{-1}$ | 0.39 [a] | 0.9475 |
| Pseudo second order | $K''_{ad} = 0.708\ (g\ mg^{-1}\ min^{-1})$. | 0.55 [b] | 0.9895 |

[a] value calculated using a trial and error method with the experimental data
[b] value calculated from the linear plot.

The comparative analysis of $R^2$ values shown in Table 2 indicates that a pseudo-second order ($R^2 > 0.9895$) reaction model explains better the kinetic data for Cr(VI) sorption to lechuguilla biomass. These results suggest that a rate-limiting step may be the chemical adsorption process of Cr(VI) binding to lechuguilla biomass. This process involves an exchange of electrons between the adsorbate and the surface of the adsorbing material. Similar results were reported by Ho and McKay /30/ for Cr(VI) adsorption by leaf mould and peat.

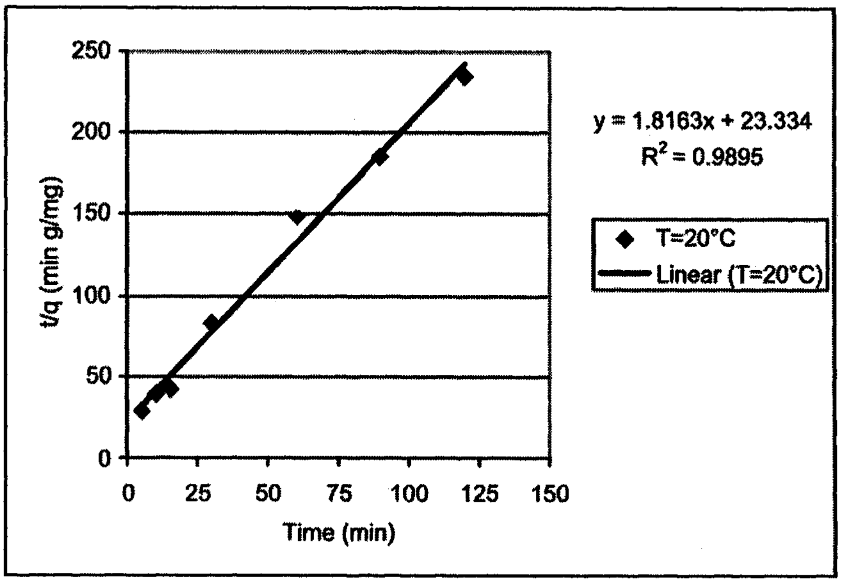

**Fig. 6:** Linear plot of pseudo second order reaction for the adsorption of Cr(VI) to lechuguilla biomass.

### 3.3 Saturation adsorption capacity

Figure 7 shows the amounts of metal accumulated (mg/g) in successive cycles of biomass contact with Cr solutions. Additionally, Table 3 shows the values for the saturation adsorption capacity and the percentage of metal recovered after 9 saturation cycles of the lechuguilla biomass. As observed in Figure 7 and column 2 of Table 3, the average q value from nine saturation cycles was 1.69 mg/g for Cr(VI) and 6.26 mg/g for Cr(III). On the other hand, the percentages of metal recovered were 42.67% and 56.23% for Cr(VI) and Cr(III),

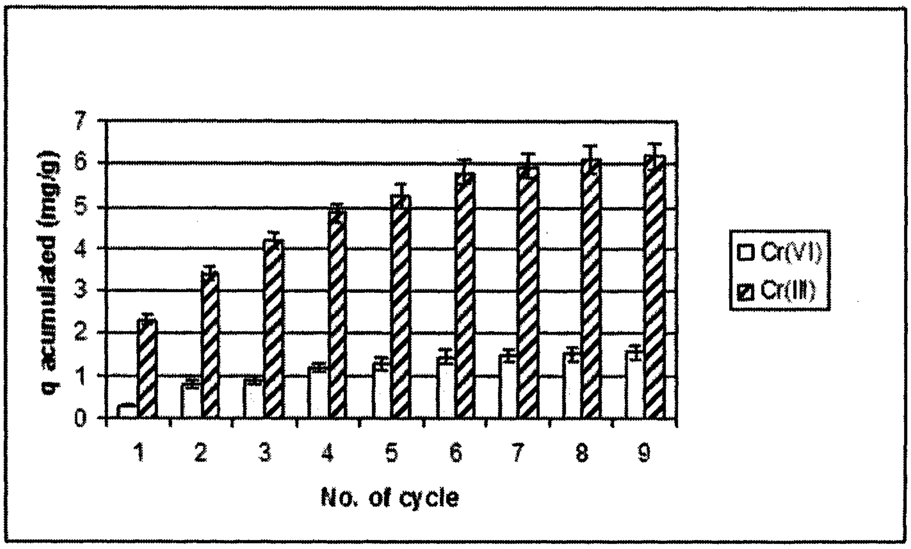

**Fig. 7:** Saturation adsorption capacity for Cr(III) and Cr(VI) by lechuguilla biomass. Experiments were performed with chromium solutions 0.3Mm concentrated. Error bars indicate 95% normalized confidence interval.

respectively (Table 3). In acidic conditions, protonation replaces the metal ions on the adsorbent surface leading to desorption of positively charged metals ions. This is further evidence that ion-exchange is involved in the adsorption mechanism of Cr(III) by lechuguilla. These results suggest that in batch conditions, lechuguilla biomass can be used up to nine times without regeneration.

**Table 3**

Result of saturation adsorption capacity for Cr(VI) and Cr(III) to lechuguilla biomass.

| Metal | $q_{saturation}$ (mg/g) | % of metal recovered |
|---|---|---|
| Cr(VI) | 1.69 | 42.67 |
| Cr(III) | 6.26 | 56.23 |

### 3.4 Isotherm models

Adsorption data is usually described by adsorption isotherms, such as Freundlich and Langmuir isotherms. These isotherms relate metal uptake per weight of adsorbent ($q_e$) to the adsorbate concentration at equilibrium ($C_e$).

The Freundlich isotherm is the most widely non-linear sorption model used. This model proposes a monolayer sorption with a heterogeneous energetic distribution of active sites, accompanied by interactions between adsorbed molecules /31/. The general form of this model is presented in equation (5):

$$q_e = K_F \, C_e^{1/n} \qquad (5)$$

where $K_F$ (mg/g) stands for adsorption capacity and $n$ for adsorption intensity.

The logarithmic form of equation (5) is:

$$\log q_e = \log K_F + 1/n \log C_e \qquad (6)$$

where $K_F$ and $1/n$ can be determined from the linear plot of log ($q_e$) versus log ($C_e$). Experimental values obtained for the adsorption capacity experiments were used to calculate the isothermal models. Table 4 shows the values of the linearized data obtained for Cr(VI) and Cr(III), respectively.

The Langmuir model represents one of the first theoretical treatments of nonlinear sorption and suggests that uptake occurs on an homogeneous surface by monolayer sorption without interaction between adsorbed molecules. In addition, the model assumes uniform energies of adsorption onto the surface and no transmigration of the adsorbate /32/. The Langmuir isotherm is given by:

$$q_e = Q_L b C_e / (1 + bC_e) \qquad (7)$$

where $Q_L$ (mg/g) and b are Langmuir constants related to adsorption capacity and the energy of adsorption, respectively. Equation (7) is usually linearized to obtain the following form:

$$1/q_e = 1/ Q_L + (1/b\, Q_L)(1/C_e) \qquad (8)$$

The linearized plots of $1/q_e$ versus $1/C_e$ for Cr(VI) and Cr(III), respectively were analyzed and the results are shown in Table 4.

**Table 4**

Comparative analysis of linear isotherms of Freundlich and Langmuir, their parameters and correlation coefficients ($R^2$ values), for the adsorption of Cr(VI) and Cr(III) to lechuguilla biomass.

| | Freundlich | | | Langmuir | | | |
|---|---|---|---|---|---|---|---|
| Metal | $K_F$ (mg/g) | *n* | $R^2$ | $Q_L$(mg/g) | b (lt/mg) | $R_L$ | $R^2$ |
| Cr(VI) | 2.04 | 13.53 | 0.9607 | 33.55 | 0.772 | 0.06 | 0.8842 |
| Cr(III) | 1.45 | 12.54 | 0.9556 | 63.69 | 0.310 | 0.135 | 0.9769 |

The adsorption intensity ( $R_L$) can be expressed as /33/:

$$R_L = 1/(1 + bC_o) \tag{9}$$

where $C_o$ (mg/l) is the initial concentration of the metal. If the average of the $R_L$ values for each of the different initial concentrations used is between 0 and 1, it indicates favorable adsorption.

The values shown in Table 4 indicate that the adsorption pattern for Cr(VI) on lechuguilla followed the Freundlich isotherm ($R^2$ > 0.9607 vs. $R^2$ >0.8842), while the adsorption pattern for Cr(III) followed the Langmuir isotherm ($R^2$ > 0.9767 vs. $R^2$ >0.9556). The values obtained for Cr(VI) from the Freundlich model show an adsorption capacity ($K_F$) of 2.04 mg/g and an affinity value (*n*) equal to 13.53, which represents a very favorable adsorption of Cr(VI). According to the Langmuir model the Cr(III) adsorption capacity ($Q_L$) of lechuguilla was 63.69 mg/g, with an affinity ($R_L$) of 0.135, which suggests a favorable Cr(III) adsorption. The adsorption capacities showed by lechuguilla biomass are slightly higher than the average values obtained with similar experimental conditions. Typical values found in literature are $0.4 < K_F < 2.7$ mg/g for Cr(VI) and $1.4 < Q_L < 119$ mg/g for Cr(III) /16, 34/. It is important to indicate that the saturation adsorption capacity, $q_{saturation}$ (mg/g), and adsorption capacity, $K_F$ or $Q_L$ (mg/g) from isotherm models, are different because the first indicates the maximum amount of metal binding in transient state and the second indicates the maximum amount of metal binding in condition of equilibrium.

### 4. CONCLUSION

The results of this investigation demonstrated that lechuguilla biomass has the potential for the removal of trivalent and hexavalent chromium from aqueous solution. Although the maxima adsorption capacities were obtained at pH 4 (95%) and pH 2 (34%) for Cr(III) and Cr(VI), respectively, the experimental data showed that even at pH 6 the biomass bound a certain amount of Cr(VI). The saturation adsorption capacities and the percentage of metal recovery were higher for Cr(III) than for Cr(VI). The adsorption process of Cr(VI) by lechuguilla was found to be time-dependent, but that of Cr(III) adsorption was not. A pseudo-second order reaction model best described the kinetic data for the adsorption of Cr(VI). Adsorption isotherms showed that the adsorption pattern for Cr(VI) followed the Freundlich isotherm, while that for Cr(III) followed the Langmuir isotherm. The adsorption capacity showed by lechuguilla was slightly higher than the average values reported in the literature.

### ACKNOWLEDGEMENTS

The authors would like to acknowledge the financial support from the National Institutes of Health (NIH) (Grant S06GM8012-33) and the University of Texas at El Paso's Center for Environmental Resource Management (Cooperative agreement CR-819849-01-04) through funding from the Office of Exploratory Research of the EPA. In addition, the authors acknowledge the financial assistance from HBCU/MI ETC that is funded by the Department of Energy. Dr. Gardea-Torresdey acknowledges the funding from the National Institute of Environmental Health Sciences (Grant R01ES11367-01). Jaime Romero-González also acknowledges the financial support from the University of Guanajuato, Mexico.

### REFERENCES

1. C. Pellerin and S. M. Booker, *Environ. Health Perspect*, **108**, 12 (2000)
2. K. R. Reddy, U. S. Parupudi, S. N. Devulapalli and C. Y. Xu, *J. Hazard. Mater*, **55**, 135 (1997)
3. WHO, *Health Criteria 61, Chromium,* World Health Organization: Geneva (1988)
4. J. Kotaś, Z. Stasicka, *Environ. Pollut*, **107**, 263 (2000)
5. C. P. Jordão, J. L. Pereira and G. N. Jham, *Sci. Total Environ.* **207**, 1 (1997)
6. US EPA, *National Primary Drinking Water Regulations,* United States Environmental Protection Agency, Sec **141.2** , 93 (1992)
7. C PHG, *Public Health Goal for Chromium in Drinking Water,* California Public Health Goal, California (1999)
8. WHO, *Guidelines for Drinking-Water Quality*, World Health Organization, **1**, 45 (1993)
9. S. Kocaoba and G. Akcin, *Talanta*, **57**, 23 (2002)
10. I. Gaballah and G. Kilbertus, *J. Geochem. Explor*, **62**, 241 (1998)
11. N. Daneshvar, D. Salari and S. Aber, *J. Hazard. Mater*, **B94**, 49 (2002)
12. Z. Aksu, Ü. Açikel, E. Kabasakal and S. Tezer, *Water Res*, **36**, 3063 (2002)
13. Y. Guo, J. Qi, Y. Shaofeng, K. Yu, Z. Wang and H. Xu, *Mater.* Chem. Phys, **78**, 132 (2002)
14. J. G. Parsons, M. Hejazi, K. J. Tiemann, J. Henning and J. L. Gardea-Torresdey, *Microchem.* J, **71**, 211 (2002)
15. Z. Aksu, F. Gönen and Z. Demircan, *Process Biochem*, **00**, 1 (2002)
16. Y. Yun, D.Park, J. M. Park and B. Volesky, *Environ. Sci. Technol*, **35**, 4353 (2001)
17. N. K. Hamadi, X. D. Chen, M. M. Farid and M. G. Lu, *Chem. Eng*, J, **84**, 95 (2001)
18. S. B. Bailey, T. J. Olin, R. M. Bricka and D. D. Adrian, *Water Res*, **33**, 2469 (1999)
19. J.L. Gardea-Torresdey, K. J. Tiemann, V. Armendariz, L. Bess-Oberto, R.R. Chianelli,. J. Rios, J.G Parsons and G. Gamez, *J. Hazard. Mater*, **B80**, 175 (2000)
20. D. Kratochivil, P. Pimentel and B. Volesky, *Environ. Sci. Technol*, **32**, 2698 (1998)
21. H. S. Gentry, *Agaves of Continental North America*, The University of Arizona Press, Arizona, 1982
22. J. L. Gardea-Torresdey, K. J. Tiemann, J. H. Gonzalez, O. Rodríguez and G. Gamez, *J. Hazard. Mater*, **57**, 29 (1998)
23. G. Cimino, A. Passerini and G. Toscano, *Water Res*, **34**, 2955 (2000)
24. G. Carrillo-Morales, M. M. Dávila-Jiménez, M.P. Elizalde-González, A. A. Paláez-Cid, *J. Chromatogr. A*, **938**, 237 (2001)
25. M. Dakiky, M. Khamis, A. Manassra and M. Mer'eb, *Adv. Environ. Res*, **6**, 533 (2002)
26. E. Fourest and B. Volesky, *Biochem. Biotechnol*, **67**, 215 (1997)
27. D. Chvedov, S. Ostap and T. Le, *Colloids Surf*, **182A**, 131 (2001)

28. S. Langergren, *Handlingar*, **24**, 1(1898)
29. C. Namasivayam and K. Ranganathan, *Ind. J. Chem. Technol*, **1**, 351(1995)
30. Y. S. Ho and G. McKay, *Process Biochem*, **34**, 451 (1999)
31. H. Freundlich, *Z. Phys. Chem*, **57**, 384 (1906)
32. I. Langmuir I, *J. Am. Chem. Soc*, **40**, 1361(1918)
33. T. W. Weber and R. K. Chakraborti, *J. Am. Inst. Chem. Eng*, **20**, 228 (1974)
34. C. Namasivayam and R. T. Yamuna, *Chemosphere*, **30**, 541 (1995)

